# A real-world pharmacovigilance study of efgartigimod alfa in the FDA adverse event reporting system database

**DOI:** 10.3389/fphar.2025.1510992

**Published:** 2025-04-16

**Authors:** Yunlin Yang, Jinfeng Liu, Wei Wei

**Affiliations:** ^1^ Department of Clinical Pharmacy, Shifang People’s Hospital, Shifang, Sichuan, China; ^2^ Department of Pharmacy, People’s Hospital of Zhongjiang County, Deyang, Sichuan, China; ^3^ Department of Pharmacy and Evidence-Based Pharmacy Center, West China Second University Hospital, Chengdu, China; ^4^ Key Laboratory of Birth Defects and Related Diseases of Women and Children (Sichuan University), Ministry of Education, Chengdu, Sichuan, China

**Keywords:** adverse event, data mining, FAERS, pharmacovigilance, efgartigimod alfa, generalized myasthenia gravis

## Abstract

**Objective:**

Efgartigimod alfa, approved for treating generalized myasthenia gravis (gMG) in adult patients who are anti-acetylcholine receptor (AChR) antibody positive, has uncertain long-term safety in large populations This study analyzed adverse events (AEs) linked to efgartigimod alfa using data from the FDA Adverse Event Reporting System (FAERS).

**Methods:**

We collected and analyzed efgartigimod alfa-related reports from the FAERS database from the first quarter of 2022 through the second quarter of 2024. Disproportionality analysis was used in data mining to quantify efgartigimod alfa-related AE signals.

**Results:**

A total of 3,040 reports with efgartigimod alfa as the primary suspect and 12,487 AEs were retrieved from FAERS. The most frequently reported serious outcome was hospitalization (53.22%), and death occurred in 270 cases (8.88%). Disproportionality analysis detected 137 AE signals, with the most common in nervous system disorders (22.69%), general disorders and administration site conditions (16.90%), and infections and infestations (14.05%). Notably, in addition to infection-related AEs identified during clinical trials, this study detected unexpected signals, including inappropriate schedule of product administration (ROR 2.60, PRR 2.53, IC 1.34, EBGM 2.53) and nephrolithiasis (ROR 8.13, PRR 7.99, IC 2.99, EBGM 7.95). The median onset time of AEs was 81.0 days.

**Conclusion:**

Our study provides a comprehensive assessment of the post-marketing safety of efgartigimod alfa and highlights the need for continued vigilance regarding infection-related adverse events. Additionally, the detection of inappropriate schedules of product administration underscores the importance of enhanced training and pharmacist involvement in medication management. Further research is warranted to explore the potential association between efgartigimod alfa and nephrolithiasis.

## 1 Introduction

Generalized myasthenia gravis (gMG) is a rare chronic autoimmune neuromuscular disease (Gilhus, 2016; Beladakere Ramaswamy et al., 2021). The disease is characterized by generalized skeletal muscle weakness and exercise-induced weakness, which can have a significant negative impact on quality of life. gMG is treated with the goal of achieving complete remission, pharmacologic remission, or mild symptomatic status, while reducing adverse events (AEs) (Lascano and Lalive, 2021). Symptomatic therapy, such as acetylcholinesterase inhibitors, short-term salvage immunotherapy, such as plasma exchange and intravenous immunoglobulin, and long-term immunotherapy, such as corticosteroids and nonsteroidal immunosuppressants, comprise the standard treatment regimen for gMG (Lascano and Lalive, 2021; Menon and Bril, 2022; Habib et al., 2020). The symptoms of many patients can be effectively controlled with broad-spectrum nonspecific immunosuppressive drugs like corticosteroids, azathioprine, cyclosporine, mycophenolate mofetil, and tacrolimus; however, 10%–20% of patients are resistant or intolerant to these drugs, and many patients do not experience a complete or stable remission. Moreover, broad-spectrum nonspecific immunosuppressive drugs may need weeks to months to manifest their effects and are frequently linked to significant side effects (Alhaidar et al., 2022; Vanoli and Mantegazza, 2022).

Intravenous efgartigimod alfa is the inaugural neonatal Fc receptor (FcRn) antagonist authorized for the treatment of gMG (Heo, 2022). As one of several new targeted therapies, efgartigimod alfa is fast-acting, well-tolerated, and has the potential to provide sustained disease control in patients with gMG (Beladakere Ramaswamy et al., 2021; Menon and Bril, 2022; Habib et al., 2020). Clinical trials indicate that efgartigimod alfa is generally well tolerated in individuals with gMG, with most adverse responses classified as mild to moderate in intensity (Howard et al., 2021; Jacob et al., 2022). The most prevalent adverse events include headaches, upper respiratory tract infections, and urinary tract infections (Heo, 2022).

However, clinical trials generally have limited sample sizes and do not fully reflect the safety of efgartigimod alfa in real-world post-marketing applications. The FDA Adverse Event Reporting System (FAERS) database, on the other hand, is an invaluable resource for post-market monitoring and identification of drug safety issues (Feng et al., 2022), utilizing disproportionality analysis in a database of spontaneous reports of adverse drug events is an effective quantitative method for pharmacovigilance signal detection (Cutroneo et al., 2024).

Disproportionality analysis is a method employed to formulate hypotheses on potential causal associations between a medication and an adverse event (Tyagi and Kumar, 2024). Signal detection involves reviewing individual case safety reports and conducting statistical analyses while considering the nature of the data, its characteristics, and the type of drug (Jain et al., 2023). Multiple reports with high-quality information are needed to produce a signal (Javed and Kumar, 2024; Sharma et al., 2023). This study offers a thorough evaluation of the safety of egartigimod alfa in real-world settings through a detailed review of FAERS data, enhancing clinician knowledge and fostering safer medication utilization.

## 2 Materials and methods

### 2.1 Data source

**FIGURE 1:**
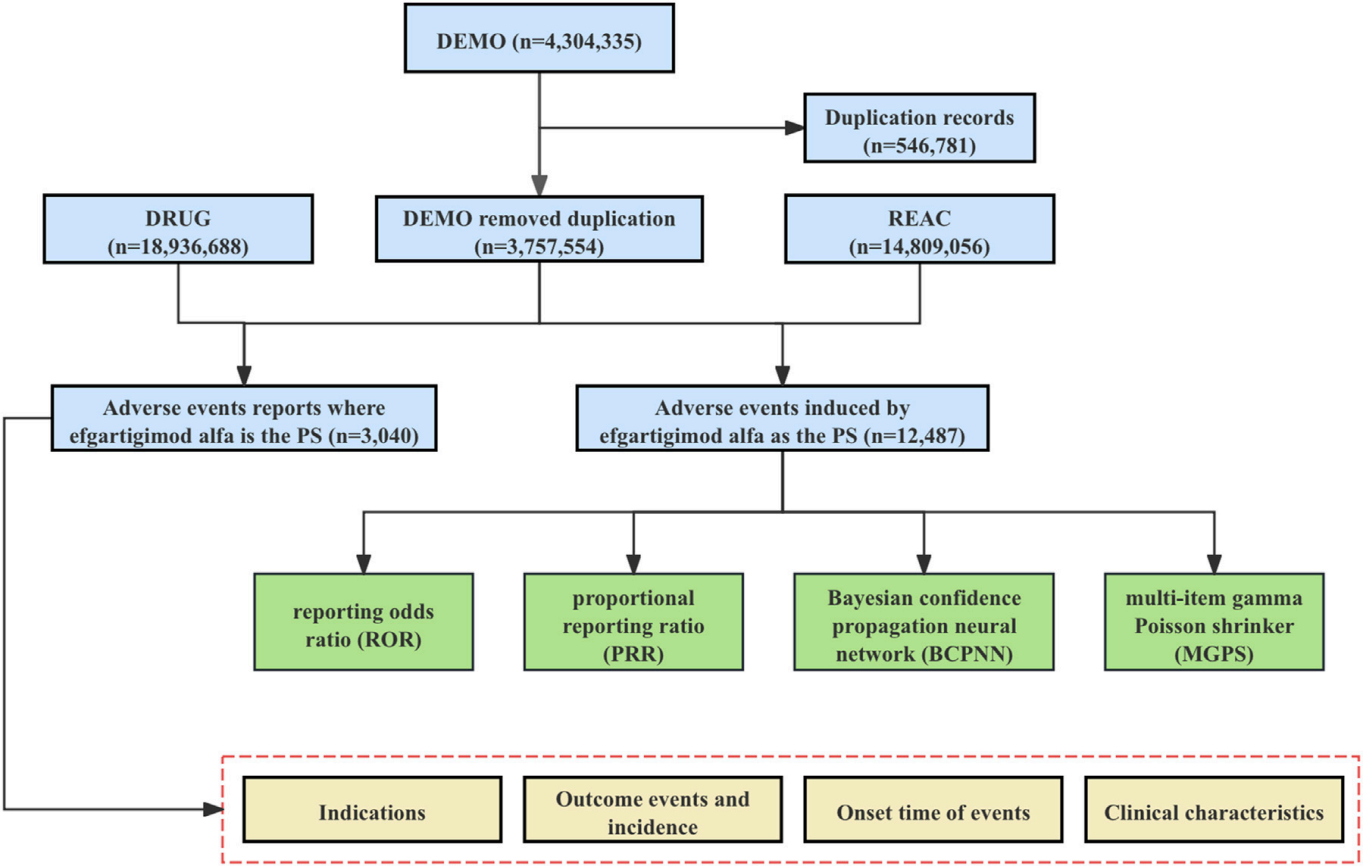
The flowchart for identifying efgartigimod alfa AEs in the FAERS database. Abbreviations: FAERS, United States Food and Drug Administration Adverse Event Reporting System; DEMO, demographic and administrative information file; DRUG, drug information file; REAC, adverse events file; PS, Primary Suspect.

We conducted a retrospective pharmacovigilance study using data from the FAERS database covering the first quarter of 2022 through the second quarter of 2024. The FAERS data file consists of seven types of datasets: patient demographics and management information (DEMO), drug/biologic information (DRUG), adverse events (REAC), patient outcomes (OUTC), reporting source (RPSR), start and end dates of drug therapy (THER), and indications for use/diagnosis (INDI). We downloaded each quarter’s data from the FDA website [FAERS Quarterly Data Extract Files (www.fda.gov)] to import into MySQL for analysis. We obtained a total of 4,304,335 AE reports during the time period identified in this study. Due to the presence of duplicate reports, we removed the duplicates before performing further data analysis by selecting the most recent FDA_DT when the CASEID was the same, and selecting the larger PRIMARYID when the CASEID and FDA_DT were the same, as per FDA recommendations, resulting in 3,757,554 AE reports (Figure 1).

### 2.2 Adverse event and drug identification

FAERS is a database that contains information on adverse events and medication error reports submitted to the FDA (Rodriguez et al., 2001; Wysowski and Swartz, 2005). In addition to reports from manufacturers, healthcare professionals and the public can also submit reports. The FAERS structure follows the International Safety Reporting Guidelines ICH E2B issued by the International Conference on Harmonization (ICH) (Lomeli-Silva et al., 2024). AEs are coded as terms in the Medical Dictionary for Regulatory Activities (MedDRA) (Brown et al., 1999). MedDRA categorizes words into five hierarchical levels: System Organ Class (SOC), High-Level Group terms (HLGT), High-Level Terms (HLT), Preferred Term (PT), and Low Level Terms (LLT), enhancing data organization and searchability across different tiers (Omar et al., 2021). This study utilized MedDRA version 27.1, the most recent iteration at the time of analysis. FAERS allows reporting of any FDA-approved drug, and the drug names in this study were the generic and trade names, including efgartigimod, VYVGART, and VYVGART HYTRULO, respectively. To enhance the precision of the analysis, we chose to report only AEs for which efgartigimod alfa was the primary suspect (PS) drug for inclusion in this study.

### 2.3 Data mining

Disproportionality analysis is a basic analytical method used in pharmacovigilance studies that compares the proportion of the target drug that undergoes a specific AE with all other drugs (Hu et al., 2020). An AE signal is deemed created when the occurrence of a certain adverse event associated with a particular medicine substantially exceeds the background frequency in the database and beyond a defined threshold. In this study, frequentist methods [reporting odds ratio (ROR) (van Puijenbroek et al., 2002) and proportional reporting ratio (PRR) (Evans et al., 2001)], Bayesian methods [information component (IC) (Bate et al., 1998) and empirical Bayes geometric mean (EBGM) (Szarfman et al., 2002)] of disproportionality analysis were applied to identify the potential AE signals associated with efgartigimod alfa. Each of these methods has distinct advantages: ROR and PRR are widely recognized and easily interpretable methods for disproportionality analysis, while IC and EBGM adjust for variability in the reporting rates and offer more robust estimates for signals where data might be sparse. In order to improve the accuracy of the analysis, the four algorithms mentioned above were only considered to satisfy the thresholds simultaneously when they produce a meaningful AE signal. Formulae and threshold conditions for the four methods are shown in Table 1.

**TABLE 1:** Four major algorithms used for signal detection.

Algorithms	Equation	Criteria
ROR	ROR=ad/b/c	lower limit of 95% CI > 1, N ≥ 3
	$95\%\text{ CI}=e^{{\ln\left(\text{ROR}\right)\pm 1.96\left(1/a+1/b+1/c+1/d\right)}^{0.5}}$	
PRR	$\text{PRR}=a\;\left(c+d\right)/c/\left(a+b\right)$	PRR ≥ 2, *χ* ^2^ ≥ 4, N ≥ 3
	$\chi^{2}=\left[{\left(\text{ad}‐\text{bc}\right)}^{2}\right]\left(a+b+c+d\right)/\left[\left(a+b\right)\;\left(c+d\right)\;\left(a+c\right)\;\left(b+d\right)\right]$	
BCPNN	$\text{IC}=\log_{2}a\left(a+b+c+d\right)\;\left(a+c\right)\;\left(a+b\right)$	IC025 > 0
	$95\%\text{ CI}=E\;\left(\text{IC}\right)\;\pm \;2V{\left(\text{IC}\right)}^{0.5}$	
MGPS	$\text{EBGM}=a\;\left(a+b+c+d\right)/\left(a+c\right)/\left(a+b\right)$	EBGM05 > 2
	$95\%\text{ CI}=e^{\ln\left(\text{EBGM}\right)\pm 1.96{\left(1/a+1/b+1/c+1/d\right)}^{0.5}}$	

Equation: a, number of reports containing both the target drug and target adverse drug reaction; b, number of reports containing other adverse drug reaction of the target drug; c, number of reports containing the target adverse drug reaction of other drugs; d, number of reports containing other drugs and other adverse drug reactions. 95% CI, 95% confidence interval; N, the number of reports; χ^2^, chi-squared; IC, information component; IC025, the lower limit of 95% CI of the IC; E (IC), the IC expectations; V(IC), the variance of IC; EBGM, empirical Bayesian geometric mean; EBGM05, the lower limit of 95% CI of EBGM.

In addition, time to AE and the proportion of serious outcomes were calculated in this study. Time to AE was defined as the interval between EVENT_DT (date of AE occurrence) and START_DT (date of initiation of treatment with efgartigimod alfa). We excluded reports with reporting errors (EVENT_DT before START_DT), inaccurate dates, or missing entries. Furthermore, we tallied the instances with serious outcomes and then divided them by the overall number of reports to get the ratio of serious outcomes. All data processing was performed using MYSQL 8.0, Navicat Premium 16, and Microsoft Excel 2021.

### 2.4 Subgroups analysis

A subgroup analysis was conducted to determine differences in adverse event signals associated with efgartigimod alfa among specific populations. The analysis was stratified by gender (i.e., male and female).

### 2.5 Sensitivity analysis

To assess the impact of concomitant medications on the observed outcomes, we conducted a sensitivity analysis focusing on the newly detected AE signals. Specifically, we excluded AE reports that involved the concurrent administration of other medications. This approach allowed us to determine whether the inclusion of these reports significantly influenced our results. The findings from the sensitivity analysis were compared to those of the primary analysis to evaluate the robustness of our study conclusions.

## 3 Results

### 3.1 Descriptive analysis

Excluding duplicate reports, we retrieved a total of 3,040 reports of efgartigimod alfa as PS and 12,487 cases of AEs induced by efgartigimod alfa from the first quarter of 2022 to the second quarter of 2024. After the drug was introduced to the market, there was a trend of increasing AE reports year by year, and the total number of reports in the first two quarters of 2024 has exceeded that of all of 2023. There was a significant amount of missing data in the age and sex fields in all reports, with 2,266 cases (75.54%) not reporting sex. In addition to this, patient age was not reported for 2,783 cases (91.55%). The vast majority of reports came from the United States (85.99%), followed by Japan (5.59%) and Germany (1.71%). Consumers submitted 79.54% of the reports and healthcare professionals submitted 19.34% of the reports. Myasthenia gravis was the most reported indication (68.52%), and as for reported serious outcomes, hospitalization was the most reported (53.22%), followed by other serious (important medical event) with 47.63%. In addition, death was reported in 270 cases (8.88%). For details, see Table 2.

**TABLE 2:** Clinical characteristics of reports with efgartigimod alfa from the FAERS database (January 2022 to June 2024).

Characteristics	Subgroups	Case number, n	Case proportion, %
Number of events		3,040	
Gender	Female	402	13.22
	Male	372	12.24
	Unknown	2,266	75.54
Age	<18 years	1	0.03
	18–44 years	34	1.12
	45–64 years	74	2.43
	≥65 years	148	4.87
	Unknown	2,783	91.55
Reporter	Consumer	2,418	79.54
	Health Professional	263	8.65
	Physician	263	8.65
	Pharmacist	62	2.04
	Unknown	34	1.12
Reported Countries	America	2,614	85.99
	Japan	170	5.59
	Germany	52	1.71
	Great Britain	32	1.05
	Canada	14	0.46
	Country not specified or others	158	5.20
Year	2024 q1-q2	1,468	48.29
	2023	1,227	40.36
	2022	345	11.35
Indications	Myasthenia gravis	2,083	68.52
	Immune thrombocytopenia	12	0.40
	Pemphigus	6	0.20
	Chronic inflammatory demyelinating polyradiculoneuropathy	4	0.13
	Muscular weakness	4	0.13
	Others and Unknown	931	30.63
Serious Outcome	Hospitalization	1,618	53.22
	Other Serious (Important Medical Event)	1,448	47.63
	Death	270	8.88
	Life-Threatening	174	5.72
	Required intervention to prevent permanent inmairment/damage	8	0.26
	Disability	7	0.23

Abbreviations: FAERS, United States Food and Drug Administration Adverse Event Reporting System; q1, quarter 1; q2, quarter 2.

### 3.2 Time to onset of efgartigimod alfa-associated adverse events

**FIGURE 2:**
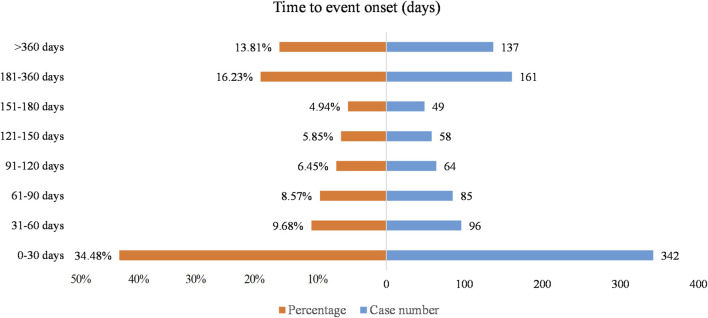
Time-to-onset of efgartigimod alfa-associated AEs.

Excluding erroneous, absent, or unidentified reports of adverse event, a total of 992 AEs documented the time of commencement, with a median onset time of 81.0 days (interquartile range 15.0–220.5 days). As shown in Figure 2, efgartigimod alfa resulted in the greatest number of AEs occurring in the first 1 month after initiation of therapy (342 cases, 34.48%), and in addition, 13.81% (137 cases) of AEs occurred after 1 year of dosing.

### 3.3 Disproportionality analysis

**FIGURE 3:**
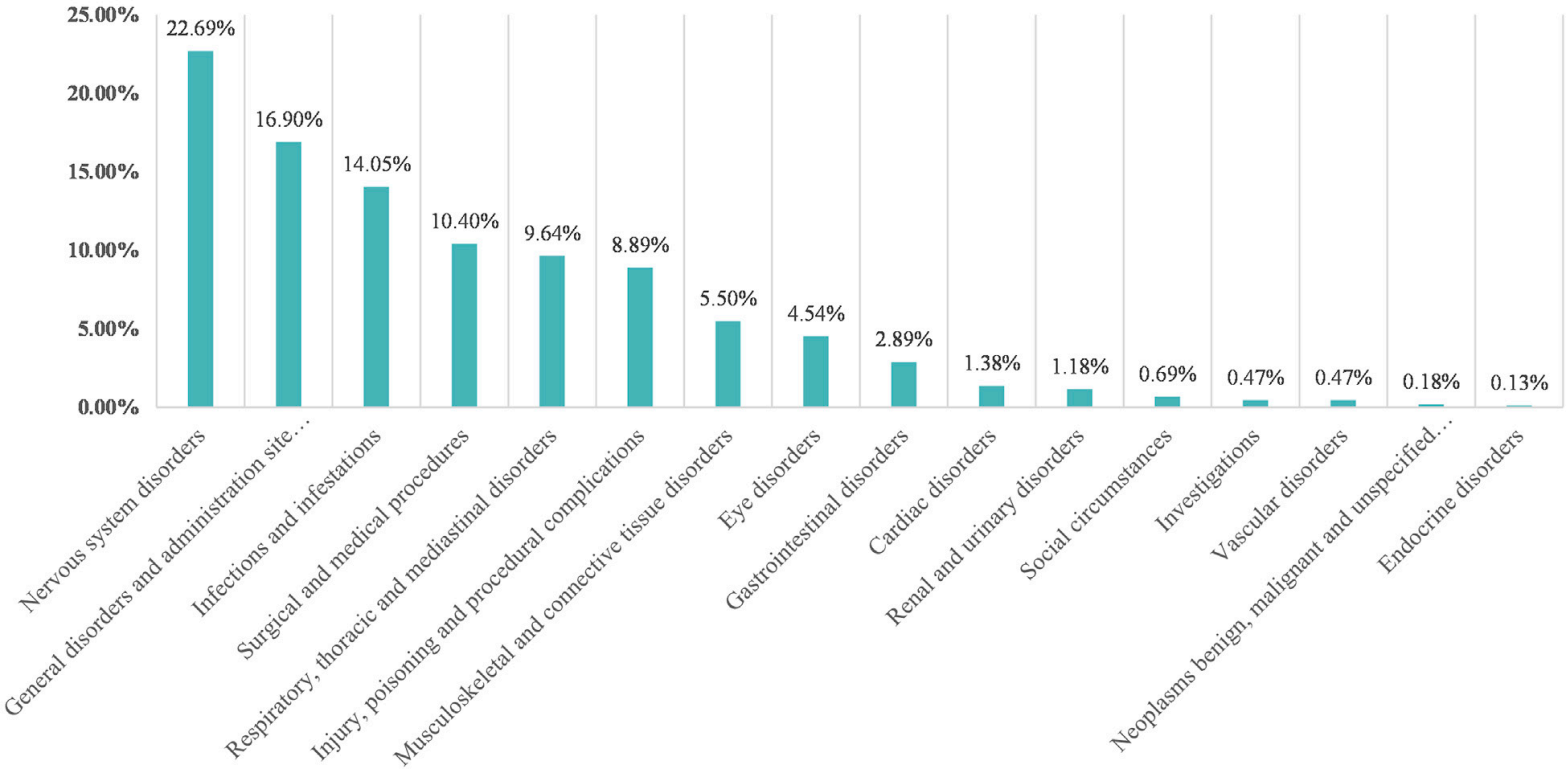
Proportion of efgartigimod alfa-associated AEs in different organ systems.

Figure 3 shows the percentage of AEs in each SOC classification. Efgartigimod alfa caused the most common AEs in nervous system disorders (22.69%), followed by general disorders and administration site conditions (16.90%) and infections and infestations (14.05%). A total of 137 AE signals were detected in 16 SOCs. The FAERS database collects information on all medical and healthcare-related AEs, so we excluded some AE signals that were related to the patient’s primary disease and those that were not related to medication use, as outlined in Supplementary Table S1. Table 3 shows the number of AE signals detected at the AE signals detected at the preferred term (PT) level. In this study, the SOC detected a total of 20 AE signals: infection and invasion. This coincides with warnings and precautions on drug labels. The three AEs with the highest reporting rate were urinary tract infection (180 cases), pneumonia (130 cases), and nasopharyngitis (84 cases). In addition to this, we found some important and noteworthy AE signals. Inappropriate schedule of product administration was reported in 131 cases with signal intensities of ROR 2.60 (2.18–3.10), PRR 2.53 (122.93), IC 1.34 (1.05), and EBGM 2.53 (2.12). Another 58 cases reported nephrolithiasis with signal intensity of ROR 8.13 (6.26–10.55), PRR 7.99 (353.46), IC 2.99 (2.42), and EBGM 7.95 (6.12).

**TABLE 3:** Signal strength of reports of efgartigimod alfa at the Preferred Term (PT) level in the FAERS database.

SOC	Preferred terms (PTs)	Efgartigimod alfa cases reporting PT	ROR (95% two-sided CI)	PRR (*χ* ^2^)	IC (IC025)	EBGM (EBGM05)
Vascular disorders	Poor venous access	26	9.81 (6.66–14.45)	9.73 (202.33)	3.27 (2.26)	9.67 (6.56)
Cardiac disorders	Atrial fibrillation	56	4.24 (3.25–5.53)	4.18 (135.70)	2.06 (1.57)	4.17 (3.20)
	Cardiac failure congestive	20	4.12 (2.65–6.40)	4.10 (46.73)	2.03 (1.13)	4.09 (2.63)
Gastrointestinal disorders	Dysphagia	139	11.53 (9.71–13.67)	11.04 (1263.74)	3.45 (3.09)	10.96 (9.23)
	Oesophageal stenosis	4	11.29 (4.22–30.24)	11.28 (37.14)	3.48 (0.27)	11.19 (4.18)
	Salivary hypersecretion	11	7.91 (4.37–14.33)	7.89 (65.79)	2.97 (1.36)	7.85 (4.33)
	Tongue disorder	5	6.73 (2.79–16.22)	6.72 (24.22)	2.74 (0.34)	6.69 (2.78)
Renal and urinary disorders	Nephrolithiasis^a^	58	8.13 (6.26–10.55)	7.99 (353.46)	2.99 (2.42)	7.95 (6.12)
	Micturition urgency	7	4.72 (2.25–9.93)	4.71 (20.41)	2.23 (0.47)	4.70 (2.24)
General disorders and administration site conditions	Symptom recurrence	152	70.70 (59.81–83.57)	67.21 (9410.28)	6.00 (5.25)	63.8 (53.97)
	Therapeutic product ineffective	21	19.31 (12.53–29.76)	19.18 (356.54)	4.24 (2.70)	18.91 (12.27)
	Pre-existing condition improved	7	11.58 (5.50–24.4)	11.56 (66.90)	3.52 (1.10)	11.46 (5.44)
	Infusion site extravasation	10	7.73 (4.15–14.41)	7.71 (58.05)	2.94 (1.25)	7.67 (4.11)
	Therapeutic response shortened	64	6.72 (5.24–8.61)	6.60 (303.15)	2.71 (2.21)	6.57 (5.12)
	Therapy non-responder	58	6.24 (4.81–8.10)	6.14 (249.12)	2.61 (2.09)	6.11 (4.71)
	Drug effect less than expected	27	5.72 (3.91–8.36)	5.67 (103.64)	2.50 (1.68)	5.65 (3.87)
	Asthenia	213	4.37 (3.80–5.03)	4.14 (513.81)	2.05 (1.81)	4.13 (3.59)
	Therapeutic product effect decreased	47	3.87 (2.90–5.16)	3.82 (98.08)	1.93 (1.40)	3.81 (2.86)
	Fatigue	332	2.84 (2.54–3.19)	2.64 (353.05)	1.40 (1.21)	2.64 (2.36)
Endocrine disorders	Thyroid mass	7	10.29 (4.89–21.67)	10.27 (58.08)	3.35 (1.03)	10.19 (4.84)
Neoplasms benign, malignant and unspecified (incl cysts and polyps)	Thymoma	10	213.64 (109.10–418.36)	212.94 (1799.25)	7.51 (2.39)	181.77 (92.82)
Musculoskeletal and connective tissue disorders	Mastication disorder	26	71.65 (48.18–106.55)	71.04 (1698.01)	6.07 (3.67)	67.23 (45.21)
	Muscle fatigue	7	11.72 (5.56–24.7)	11.70 (67.86)	3.54 (1.11)	11.60 (5.51)
	Muscular weakness	153	11.01 (9.35–12.96)	10.50 (1310.86)	3.38 (3.04)	10.42 (8.85)
	Jaw disorder	4	10.18 (3.80–27.24)	10.16 (32.79)	3.33 (0.23)	10.09 (3.77)
	Muscle twitching	15	5.41 (3.25–8.99)	5.39 (53.38)	2.42 (1.26)	5.37 (3.23)
	Musculoskeletal chest pain	10	4.56 (2.45–8.49)	4.55 (27.57)	2.18 (0.77)	4.53 (2.43)
	Back pain	88	2.73 (2.21–3.38)	2.68 (93.70)	1.42 (1.06)	2.68 (2.17)
Injury, poisoning and procedural complications	Inappropriate schedule of product^a^ administration	131	2.60 (2.18–3.10)	2.53 (122.93)	1.34 (1.05)	2.53 (2.12)
Nervous system disorders	Bulbar palsy	11	368.50 (187.81–723.06)	367.17 (3096.43)	8.15 (2.60)	283.26 (144.36)
	Dysarthria	66	16.94 (13.25–21.65)	16.59 (955.44)	4.03 (3.36)	16.38 (12.82)
	Amyotrophic lateral sclerosis	4	15.85 (5.91–42.53)	15.83 (54.89)	3.97 (0.38)	15.65 (5.83)
	Tunnel vision	4	14.13 (5.27–37.89)	14.11 (48.20)	3.80 (0.35)	13.97 (5.21)
	Facial paresis	5	13.71 (5.68–33.13)	13.69 (58.19)	3.76 (0.69)	13.55 (5.61)
	Drooling	9	10.67 (5.53–20.58)	10.64 (77.93)	3.40 (1.37)	10.55 (5.47)
	Spinal cord compression	4	8.08 (3.02–21.62)	8.07 (24.63)	3.00 (0.13)	8.03 (3.00)
	Speech disorder	34	5.43 (3.87–7.62)	5.38 (121.06)	2.42 (1.72)	5.36 (3.82)
	Facial paralysis	7	5.05 (2.40–10.62)	5.04 (22.59)	2.33 (0.53)	5.02 (2.39)
	Hemiparesis	8	4.69 (2.34–9.41)	4.68 (23.11)	2.22 (0.60)	4.67 (2.33)
	Dysstasia	18	4.32 (2.72–6.88)	4.30 (45.56)	2.10 (1.13)	4.29 (2.70)
	Balance disorder	45	4.18 (3.11–5.62)	4.14 (107.02)	2.04 (1.49)	4.13 (3.07)
Infections and infestations	Herpes zoster reactivation	3	30.65 (9.74–96.42)	30.62 (83.88)	4.90 (0.02)	29.9 (9.51)
	Prostate infection	4	27.95 (10.37–75.33)	27.91 (101.5)	4.77 (0.52)	27.32 (10.13)
	Epididymitis	4	27.63 (10.25–74.48)	27.6 (100.30)	4.76 (0.52)	27.02 (10.02)
	Diverticulitis	46	11.91 (8.89–15.95)	11.74 (448.41)	3.54 (2.79)	11.64 (8.69)
	Respiratory syncytial virus infection	18	8.24 (5.18–13.12)	8.20 (113.08)	3.03 (1.82)	8.15 (5.12)
	Upper respiratory tract infection	53	8.05 (6.13–10.58)	7.93 (319.70)	2.98 (2.38)	7.89 (6.01)
	Urinary tract infection	180	7.03 (6.05–8.18)	6.67 (871.44)	2.73 (2.45)	6.64 (5.71)
	Urosepsis	8	6.13 (3.06–12.29)	6.11 (34.07)	2.61 (0.83)	6.09 (3.04)
	Meningitis	5	6.10 (2.53–14.69)	6.09 (21.17)	2.60 (0.28)	6.06 (2.52)
	Herpes zoster	48	5.49 (4.13–7.31)	5.42 (172.82)	2.43 (1.86)	5.40 (4.06)
	Respiratory tract infection	21	5.18 (3.37–7.96)	5.15 (69.97)	2.36 (1.43)	5.13 (3.34)
	Cellulitis	32	4.93 (3.48–6.99)	4.89 (98.83)	2.29 (1.57)	4.87 (3.44)
	Pneumonia aspiration	15	4.57 (2.75–7.61)	4.56 (41.54)	2.18 (1.08)	4.54 (2.73)
	Sepsis	63	4.52 (3.52–5.80)	4.44 (168.25)	2.15 (1.68)	4.43 (3.45)
	Post procedural infection	6	4.49 (2.01–10.01)	4.48 (16.17)	2.16 (0.27)	4.47 (2.00)
	Staphylococcal infection	14	4.10 (2.42–6.94)	4.09 (32.56)	2.03 (0.91)	4.08 (2.41)
	Kidney infection	12	3.87 (2.19–6.82)	3.85 (25.31)	1.94 (0.74)	3.85 (2.18)
	Bronchitis	28	2.96 (2.04–4.30)	2.95 (36.01)	1.56 (0.87)	2.94 (2.03)
	Pneumonia	130	2.91 (2.44–3.48)	2.83 (156.19)	1.50 (1.21)	2.83 (2.37)
	Nasopharyngitis	84	2.67 (2.15–3.31)	2.62 (84.89)	1.39 (1.02)	2.62 (2.11)

^a^ Emerging findings of efgartigimod alfa associated AEs from FAERS database.

ROR, reporting odds ratio; PRR, proportional reporting ratio; *χ* ^2^, chi-squared; IC, information component; EBGM, empirical Bayesian geometric mean.

### 3.4 Subgroup analysis

Reports from male and female patients were collected and analyzed separately, and AE signals were calculated accordingly. Table 4 presents the distribution of AE signals based on ROR values across different genders. Overall, the number of AE signals associated with efgartigimod alfa was generally similar between male (23 signals) and female (22 signals) patients. Regarding the newly identified AE signal of nephrolithiasis, there were five reported cases in male patients with a signal strength of ROR 5.40 (2.23–13.05), PRR 5.34 (17.64), IC 2.41 (0.18), and EBGM 5.33 (2.20), while six cases were reported in female patients with a signal strength of ROR 6.12 (2.73–13.71), PRR 6.04 (25.27), IC 2.59 (0.49), and EBGM 6.03 (2.69). The signal strength of nephrolithiasis was comparable between male and female patients.

**TABLE 4:** Signal strength of reports of efgartigimod alfa at the Preferred Term (PT) level in the FAERS database based on gender.

SOC	PT	Male		Female	
		Number of reports	ROR (95% two-sided CI)	Number of reports	ROR (95% two-sided CI)
Eye disorders	Eyelid ptosis	4	35.62 (13.24–95.89)	5	23.48 (9.70–56.85)
Gastrointestinal disorders	Dysphagia	13	8.26 (4.75–14.38)	10	6.07 (3.24–11.37)
Renal and urinary disorders	Nephrolithiasis^a^	5	5.40 (2.23–14.05)	6	6.12 (2.73–13.71)
General disorders and administration site conditions	Asthenia	—	—	25	3.48 (2.23–5.22)
	Symptom recurrence	9	35.79 (18.41–69.55)	22	76.12 (49.37–117.36)
	Therapeutic product ineffective	3	32.14 (10.27–100.61)	—	—
	Therapy non-responder	—	—	9	7.06 (3.64–13.67)
	Drug effect less than expected	—	—	4	7.09 (2.65–19.01)
Respiratory, thoracic and mediastinal disorders	Dyspnoea	29	3.79 (2.59–5.53)	31	3.07 (2.14–4.41)
	Choking	13	51.54 (29.52–89.96)	12	34.23 (19.23–60.91)
	Pharyngeal swelling	—	—	5	11.10 (4.59–26.83)
Surgical and medical procedures	Hospitalisation	19	5.00 (3.15–7.93)	19	5.75 (3.63–9.12)
	Mechanical ventilation	4	117.04 (43.01–318.47)	6	294.67 (128.41–676.22)
	Endotracheal intubation	—	—	5	119.36 (48.87–291.53)
	Thymectomy	—	—	3	4679.54 (941.63–23255.53)
Musculoskeletal and connective tissue disorders	Muscular weakness	16	9.95 (6.03–16.44)	—	—
	Mastication disorder	3	71.78 (22.79–226.11)	—	—
Injury, poisoning and procedural complications	Infusion related reaction	12	12.75 (7.17–22.68)	—	—
	Procedural headache	4	2056.76 (599.51–7055.64)	4	407.93 (146.15–1138.57)
Nervous system disorders	Myasthenia gravis crisis	44	1996.24 (1369.15–2910.56)	51	2712.79 (1905.73–3861.63)
	Myasthenia gravis	24	125.06 (82.17–190.33)	32	234.97 (162.49–339.77)
	Dysarthria	7	12.73 (6.02–26.92)	—	—
	Facial paresis	3	98.78 (31.23–312.48)	—	—
	Bulbar palsy	—	—	3	638.11 (190.23–2140.50)
Infections and infestations	Urinary tract infection	12	6.23 (3.50–11.07)	29	6.19 (4.24–9.03)
	Sepsis	9	4.46 (2.30–8.65)	—	—
	Pneumonia	—	—	20	3.35 (2.14–5.27)
	Respiratory tract infection	—	—	8	12.66 (6.28–25.51)
	Cellulitis	7	8.55 (4.05–18.09)	5	5.55 (2.30–13.42)
	Diverticulitis	5	13.83 (5.71–33.49)	—	—
	Upper respiratory tract infection	4	6.81 (2.54–18.26)	—	—

^a^ Emerging findings of efgartigimod alfa associated AEs from FAERS database.

Abbreviations: ROR, reporting odds ratio.

### 3.5 Sensitivity analysis

A sensitivity analysis was performed after excluding AE reports that documented concomitant medication use. The number of reports for inappropriate schedule of product administration decreased from 131 to 126, with a signal strength of ROR 2.49 (2.09–2.98), PRR 2.43 (107.89), IC 1.28 (0.99), and EBGM 2.43 (2.03). Similarly, the number of reports for nephrolithiasis decreased from 58 to 40, with a signal strength of ROR 5.56 (4.07–7.60), PRR 5.50 (147.05), IC 2.45 (1.81), and EBGM 5.48 (4.01). Overall, although the number of reports decreased, the sensitivity analysis did not substantially alter the conclusions of the primary analysis.

## 4 Discussion

This study is the first and most extensive investigation to date of AEs related to efgartigimod alfa, using the FASRS database for post-marketing pharmacovigilance. Efgartigimod alfa is a first-of-its-kind novel human immunoglobulinG1 (IgG1) Fc fragment that binds with high affinity to the neonatal FcRn, thereby inhibiting its binding to FcRn and inhibiting its interaction with IgG. Efgartigimod alfa significantly reduces pathologic acetylcholine receptor antibodies, including serum IgG levels (Ulrichts et al., 2018) and thus exerts a therapeutic effect. A recent meta-analysis indicated that FcRn inhibitors (e.g., efgartigimod alfa) have favorable efficacy in patients with myasthenia gravis and do not carry increased safety risks (Li et al., 2024). However, further observations to determine the long-term safety of efgartigimod alfa remain critical, especially in the real-world application of the drug once it is on the market.

The warnings and precautions section of the drug labeling for efgartigimod alfa states that infections, hypersensitivity reactions, and infusion-related reactions are common AEs. This study emphasizes the importance of continuous monitoring and attention to these AEs, especially infection-related AEs, with efgartigimod alfa in the real world. In addition to this, some AEs that are not listed in the drug labeling are also cause for alarm.

The highest number of AE signals were observed in SOC: infections and infestations. The most common infections observed in pre-drug clinical trials were urinary tract infections and respiratory tract infections (Howard et al., 2021), which is consistent with the results of the present study. FcRn is associated with IgG circulation and antigen presentation in antigen-presenting cells (APC) (Baker et al., 2014; Ward and Ober, 2018) which may lead to autoimmune pathogenesis. Because IgG is one of the major immunoglobulins in the body, it is involved in neutralizing pathogens such as bacteria and viruses and removing pathogens through activation of the complement system and conditioning. And blocking FcRn leads to a decrease in IgG levels, which in turn weakens the body’s ability to cope with infections, making patients more susceptible to infection-related diseases (Zhou and Jiang, 2023). Therefore, we recommend that the administration of efgartigimod alfa should be delayed in patients with active infections until the infection is under control. And during treatment with efgartigimod alfa, it is crucial to monitor signs and symptoms associated with the infection.

For unexpected AEs, inappropriate schedule of product administration is a particularly important AE signal for healthcare professionals to be concerned about. Such events may indicate a failure to administer the drug at the correct time or frequency during actual use, which may compromise drug efficacy and increase the risk of adverse reactions. This study reveals the potential operational risks of efgartigimod alfa in the real world after its introduction to the market. Efgartigimod alfa has a complex preparation and administration process that requires strict adherence to a timeline (Heo, 2023). Any form of inappropriate dosing, such as delayed or too frequent administration, may lead to fluctuations in the patient’s IgG levels, which in turn may affect their immunomodulatory function, thereby increasing the risk of adverse events such as infections and allergies (Zhou and Jiang, 2023). In addition, incidents of unscheduled medication administration tend to occur in healthcare settings with inadequately trained medical staff or limited resources (James, 2013; Bates et al., 1995; Reason, 2000). Therefore, during the administration of efgartigimod alfa, it is critical to enhance the training of healthcare professionals to ensure that they fully understand the administration protocols and that patients are continuously monitored. In addition, it is equally important for pharmacists to be involved in reviewing medication utilization and communicating with physicians in a timely manner throughout the course of treatment (Kaushal et al., 2008; Bladh et al., 2011).

Nephrolithiasis is one of the unexpected AEs not mentioned in the drug labeling found in this study. Nephrolithiasis is a common disease with a high incidence and recurrence rate, affecting approximately 10.6% of men and 7.1% of women in the United States, and its prevalence is comparable to that of diabetes mellitus (9.7%) (Sorokin et al., 2017; Scales et al., 2012; Ma et al., 2024). In China, the prevalence rates are 6.5% and 5.1% in men and women, respectively (Zeng et al., 2017), again not to be ignored. Currently, there is no evidence that efgartigimod alfa is associated with the development of kidney stones. However, renal adverse effects or metabolic problems may occur in the long-term treatment of patients with myasthenia gravis, especially when receiving immunosuppressive drugs or corticosteroids such as prednisone. And may lead to alterations in calcium, vitamin D, and bone metabolism, which may increase the risk of kidney stone formation (Compston et al., 2019; Oresta et al., 2021; Porter and Kaplan, 2011). Therefore, we further extracted will efgartigimod alfa as PS and reported AE reports of nephrolithiasis (Supplementary Table S2). Of these 58 reports, only nine reported prednisone as secondary suspect drug (SS) or concomitant (C), and two reported prednisone and mycophenolate mofetil as SS or C. The current study indicates that the correlation between efgartigimod alfa and nephrolithiasis is concerning, necessitating more high-quality research to validate or refute a causal link between the two.

This study still has some limitations that are worth exploring. First, the FAERS database is a self-reporting system, and due to its own limitations, there are omissions, duplicate reports, and incomplete case information, all of which may affect the results of the analysis (Sharma and Kumar, 2022). Second, even after implementing the FDA’s suggested data cleaning and de-duplication processes, there is still a possibility of encountering duplicate reports. This might potentially result in an overestimation of the intensity of some AE signals (Cutroneo et al., 2024; Schilder et al., 2023).Third, this study could not establish a cause-and-effect link between efgartigimod alfa and particular AEs due to the use of disproportionality analyses. These analyses only give an evaluation of the strength of the signal, which is merely statistically significant. To validate the findings of this investigation, more high-quality studies are required. Furthermore, the FAERS database lacks racial and ethnicity data, which are crucial for evaluating drug-related adverse events considering both ecological and genetic factors (Yu et al., 2021). Additionally, due to a substantial proportion of missing age data, we were unable to conduct further subgroup analyses to determine the differences in AE occurrence across different age groups. Although there are certain limitations, the findings of this study will serve as a significant point of reference for healthcare practitioners to attentively observe any negative events that may occur in patients receiving efgartigimod alfa treatment.

## 5 Conclusion

This pharmacovigilance research investigated AEs linked to efgartigimod alfa in the FAERS database, which is a comprehensive assessment of the long-term safety of efgartigimod alfa after it has been approved and marketed. Our findings suggest that infection-related illnesses caused by efgartigimod alfa continue to require a high degree of vigilance in real-world applications after marketing. In addition, healthcare professionals should be vigilant in recognizing and preventing inappropriate schedules of product administration, and efforts should be made to enhance related education and training. And more research is needed to clarify the causal relationship between efgartigimod alfa and nephrolithiasis.

## Data Availability

The datasets presented in this study can be found in online repositories. The names of the repository/repositories and accession number(s) can be found below: https://figshare.com/articles/dataset/Adverse_events_associated_with_efgartigimod_use/27123546?file=49460241.
