# Supplementary material for: A real-world pharmacovigilance study of efgartigimod alfa in the FDA adverse event reporting system database
Source: Front Pharmacol. 2025 Apr 16;16:1510992. doi: 10.3389/fphar.2025.1510992 (PMC12041031; doi:10.3389/fphar.2025.1510992)
Supplement: Supplementary file 1 [file Table1.docx]

| Supplementary Table 1. Signal strength of reports of efgartigimod alfa at the Preferred Term (PT) level in the FAERS database. | | | | | | |
| --- | --- | --- | --- | --- | --- | --- |
| SOC | Preferred terms (PTs) | Efgartigimod alfa cases reporting PT | ROR (95%two-sided CI) | PRR (χ2) | IC (IC025) | EBGM (EBGM05) |
| Social circumstances | Immobile | 8 | 16.29(8.10-32.75) | 16.25(113.03) | 4.00(1.46) | 16.05(7.98) |
|  | Walking aid user | 15 | 15.80(9.48-26.32) | 15.73(204.31) | 3.96(2.21) | 15.54(9.33) |
|  | Wheelchair user | 6 | 9.15(4.10-20.45) | 9.14(43.17) | 3.18(0.76) | 9.08(4.06) |
|  | Bedridden | 9 | 5.14(2.67-9.90) | 5.13(29.81) | 2.35(0.79) | 5.11(2.65) |
| Respiratory, thoracic and mediastinal disorders | Dependence on respirator | 7 | 88.42(41.04-190.51) | 88.22(563.37) | 6.36(1.68) | 82.40(38.24) |
|  | Respiratory muscle weakness | 3 | 77.27(24.05-248.20) | 77.19(212.34) | 6.18(0.11) | 72.71(22.63) |
|  | Dyspnoea at rest | 23 | 54.20(35.65-82.41) | 53.80(1142.22) | 5.69(3.40) | 51.60(33.93) |
|  | Diaphragmatic disorder | 3 | 38.63(12.24-121.97) | 38.59(106.54) | 5.23(0.04) | 37.46(11.86) |
|  | Choking | 93 | 36.78(29.83-45.34) | 35.68(3049.64) | 5.12(4.36) | 34.71(28.15) |
|  | Hypopnoea | 5 | 10.43(4.32-25.17) | 10.41(42.20) | 3.37(0.58) | 10.33(4.28) |
|  | Pulmonary congestion | 13 | 8.11(4.70-14.01) | 8.08(80.17) | 3.01(1.54) | 8.03(4.65) |
|  | Paranasal sinus discomfort | 5 | 6.60(2.74-15.90) | 6.59(23.59) | 2.71(0.33) | 6.56(2.72) |
|  | Aspiration | 8 | 6.26(3.12-12.54) | 6.24(35.05) | 2.64(0.84) | 6.22(3.10) |
|  | Dyspnoea exertional | 29 | 4.78(3.31-6.89) | 4.74(85.49) | 2.24(1.49) | 4.73(3.28) |
|  | Respiration abnormal | 6 | 4.60(2.06-10.25) | 4.59(16.78) | 2.19(0.28) | 4.58(2.05) |
|  | Respiratory disorder | 17 | 4.41(2.73-7.10) | 4.39(44.37) | 2.13(1.12) | 4.38(2.71) |
|  | Pharyngeal swelling | 11 | 3.99(2.21-7.22) | 3.98(24.50) | 1.99(0.71) | 3.97(2.20) |
|  | Dyspnoea | 253 | 3.56(3.13-4.04) | 3.34(424.99) | 1.74(1.52) | 3.34(2.93) |
|  | Productive cough | 27 | 3.33(2.28-4.87) | 3.31(43.58) | 1.73(1.01) | 3.31(2.26) |
|  | Dysphonia | 28 | 3.19(2.20-4.63) | 3.17(41.58) | 1.66(0.97) | 3.16(2.18) |
| Surgical and medical procedures | Thymectomy | 29 | 4520.12(2064.71-9895.56) | 4477.01(28059.81) | 9.92(4.33) | 968.79(442.52) |
|  | Venous angioplasty | 3 | 1854.39(309.74-11102.1) | 1852.56(2220.67) | 9.53(0.40) | 741.62(123.87) |
|  | Plasmapheresis | 8 | 180.11(85.72-378.45) | 179.64(1240.73) | 7.29(1.99) | 156.96(74.70) |
|  | Mechanical ventilation | 21 | 100.83(64.54-157.52) | 100.14(1906.67) | 6.53(3.49) | 92.7(59.34) |
|  | Positive airway pressure therapy | 6 | 62.39(27.46-141.78) | 62.27(344.37) | 5.89(1.37) | 59.33(26.11) |
|  | Tracheostomy | 5 | 28.11(11.58-68.27) | 28.07(127.63) | 4.78(0.91) | 27.47(11.31) |
|  | Endotracheal intubation | 12 | 27.40(15.45-48.60) | 27.29(297.44) | 4.74(2.25) | 26.73(15.07) |
|  | Aortic valve replacement | 4 | 26.45(9.82-71.26) | 26.42(95.78) | 4.69(0.51) | 25.89(9.61) |
|  | Cancer surgery | 4 | 24.61(9.14-66.25) | 24.58(88.71) | 4.59(0.49) | 24.12(8.96) |
|  | Treatment delayed | 14 | 23.50(13.83-39.93) | 23.40(294.64) | 4.52(2.38) | 22.98(13.53) |
|  | Cataract operation | 15 | 23.41(14.03-39.07) | 23.30(314.32) | 4.52(2.46) | 22.89(13.72) |
|  | Intervertebral disc operation | 4 | 22.59(8.40-60.76) | 22.56(80.93) | 4.47(0.48) | 22.17(8.24) |
|  | Oxygen therapy | 7 | 18.67(8.84-39.42) | 18.63(115.08) | 4.20(1.32) | 18.37(8.70) |
|  | Colostomy | 5 | 15.01(6.21-36.28) | 14.99(64.5) | 3.89(0.73) | 14.82(6.13) |
|  | Stent placement | 11 | 14.24(7.85-25.84) | 14.20(133.43) | 3.81(1.79) | 14.05(7.74) |
|  | Cardiac pacemaker insertion | 9 | 12.90(6.68-24.90) | 12.86(97.49) | 3.67(1.49) | 12.74(6.60) |
|  | Central venous catheterisation | 6 | 12.69(5.67-28.39) | 12.67(63.83) | 3.65(0.93) | 12.55(5.61) |
|  | Endodontic procedure | 4 | 11.67(4.36-31.25) | 11.65(38.59) | 3.53(0.28) | 11.55(4.31) |
|  | Therapy cessation | 82 | 10.31(8.27-12.85) | 10.05(665.06) | 3.32(2.83) | 9.98(8.01) |
|  | Sinus operation | 4 | 10.01(3.74-26.8) | 10.00(32.15) | 3.31(0.22) | 9.93(3.71) |
|  | Coronary artery bypass | 4 | 9.06(3.39-24.24) | 9.05(28.43) | 3.17(0.18) | 8.99(3.36) |
|  | Spinal fusion surgery | 6 | 8.63(3.86-19.28) | 8.62(40.12) | 3.10(0.73) | 8.56(3.83) |
|  | Gallbladder operation | 4 | 6.78(2.54-18.14) | 6.78(19.59) | 2.75(0.04) | 6.75(2.52) |
|  | Spinal operation | 19 | 6.69(4.26-10.52) | 6.66(90.93) | 2.73(1.65) | 6.63(4.22) |
|  | Hospitalisation | 164 | 5.96(5.09-6.98) | 5.69(637.16) | 2.50(2.22) | 5.67(4.84) |
|  | Eye operation | 5 | 5.33(2.21-12.84) | 5.32(17.49) | 2.41(0.19) | 5.30(2.20) |
|  | Cholecystectomy | 7 | 5.26(2.50-11.06) | 5.25(24.01) | 2.39(0.56) | 5.23(2.49) |
|  | Tooth extraction | 8 | 5.06(2.52-10.14) | 5.05(25.86) | 2.33(0.66) | 5.03(2.51) |
|  | Therapy change | 8 | 4.83(2.41-9.68) | 4.82(24.15) | 2.27(0.62) | 4.81(2.40) |
|  | Knee arthroplasty | 15 | 3.69(2.22-6.14) | 3.68(29.25) | 1.88(0.84) | 3.67(2.21) |
|  | Therapy interrupted | 80 | 3.14(2.52-3.93) | 3.09(113.54) | 1.62(1.24) | 3.08(2.47) |
| Injury, poisoning and procedural complications | Procedural headache | 58 | 1028.51(725.68-1457.71) | 1008.9(32144.00) | 9.12(5.35) | 555.74(392.11) |
|  | Procedural dizziness | 3 | 62.86(19.69-200.63) | 62.80(173.62) | 5.90(0.09) | 59.81(18.74) |
|  | Thoracic vertebral fracture | 5 | 12.70(5.26-30.67) | 12.68(53.25) | 3.65(0.66) | 12.56(5.20) |
|  | Lumbar vertebral fracture | 6 | 9.21(4.12-20.58) | 9.19(43.50) | 3.19(0.77) | 9.13(4.09) |
|  | Shoulder fracture | 4 | 7.62(2.85-20.38) | 7.61(22.84) | 2.92(0.10) | 7.57(2.83) |
|  | Face injury | 4 | 7.56(2.83-20.22) | 7.55(22.61) | 2.91(0.09) | 7.51(2.81) |
|  | Stress fracture | 4 | 7.54(2.82-20.16) | 7.53(22.52) | 2.91(0.09) | 7.49(2.80) |
|  | Back injury | 11 | 6.69(3.69-12.11) | 6.67(52.76) | 2.73(1.22) | 6.64(3.67) |
|  | Ligament rupture | 4 | 6.49(2.43-17.35) | 6.48(18.46) | 2.69(0.01) | 6.45(2.41) |
|  | Rib fracture | 16 | 5.53(3.38-9.06) | 5.51(58.87) | 2.46(1.33) | 5.49(3.36) |
|  | Concussion | 6 | 5.04(2.26-11.24) | 5.03(19.29) | 2.33(0.36) | 5.01(2.25) |
|  | Fall | 194 | 4.55(3.93-5.27) | 4.32(501.54) | 2.11(1.86) | 4.31(3.73) |
|  | Head injury | 18 | 4.32(2.71-6.87) | 4.30(45.46) | 2.10(1.13) | 4.29(2.69) |
|  | Spinal fracture | 11 | 3.83(2.11-6.92) | 3.82(22.80) | 1.93(0.66) | 3.81(2.10) |
|  | Hip fracture | 15 | 3.42(2.06-5.69) | 3.41(25.52) | 1.77(0.75) | 3.40(2.05) |
| Myasthenia gravis crisis | Myasthenia gravis crisis | 354 | 2096.58(1770.58-2482.61) | 1852.56(262079.99) | 9.53(7.75) | 741.62(626.31) |
|  | Myasthenia gravis | 681 | 603.53(547.73-665.01) | 468.56(230491.15) | 8.41(7.70) | 339.96(308.53) |
| Investigations | Catheterisation cardiac | 4 | 8.59(3.21-22.97) | 8.58(26.59) | 3.09(0.16) | 8.52(3.19) |
|  | SARS-CoV-2 test positive | 22 | 3.31(2.18-5.04) | 3.30(35.21) | 1.72(0.91) | 3.29(2.16) |
| Eye disorders | Eyelid ptosis | 92 | 67.78(54.78-83.85) | 65.75(5572.93) | 5.97(4.91) | 62.48(50.50) |
|  | Eye symptom | 3 | 30.40(9.66-95.62) | 30.37(83.17) | 4.89(0.01) | 29.66(9.43) |
|  | Diplopia | 89 | 26.69(21.57-33.03) | 25.94(2092.81) | 4.67(3.99) | 25.43(20.55) |
|  | Blepharospasm | 17 | 18.76(11.61-30.33) | 18.66(280.03) | 4.20(2.46) | 18.40(11.38) |
|  | Asthenopia | 11 | 15.21(8.39-27.60) | 15.16(143.78) | 3.91(1.83) | 14.99(8.26) |
|  | Cataract | 38 | 4.09(2.97-5.63) | 4.05(87.21) | 2.01(1.40) | 4.04(2.93) |

ROR, reporting odds ratio; CI, confidence interval; PRR, proportional reporting ratio; χ 2, chi-squared; IC, information component; EBGM, empirical Bayesian geometric mean.
