# Supplementary material for: A real-world pharmacovigilance study of efgartigimod alfa in the FDA adverse event reporting system database
Source: Front Pharmacol. 2025 Apr 16;16:1510992. doi: 10.3389/fphar.2025.1510992 (PMC12041031; doi:10.3389/fphar.2025.1510992)
Supplement: Supplementary file 2 [file Table2.docx]

Supplementary Table 2. Efgartigimod alfa as a first suspected drug and reported adverse event ‘nephrolithiasis’ as a concomitant drug.

| primaryid | fda_dt | age | age_cod | sex | e_sub | occp_cod | occr_country | role_cod | drugname |
| --- | --- | --- | --- | --- | --- | --- | --- | --- | --- |
| 213315307 | 20230713 | 64 | YR | M | Y | CN | US | PS | VYVGART |
| 213315307 | 20230713 | 64 | YR | M | Y | CN | US | SS | VYVGART |
| 214209104 | 20230607 | 64 | YR | F | Y | CN | US | PS | VYVGART |
| 214209104 | 20230607 | 64 | YR | F | Y | CN | US | SS | VYVGART |
| 216146323 | 20230927 |  |  |  | Y | CN | US | PS | VYVGART |
| 216146323 | 20230927 |  |  |  | Y | CN | US | SS | VYVGART |
| 216146323 | 20230927 |  |  |  | Y | CN | US | SS | VYVGART |
| 216146323 | 20230927 |  |  |  | Y | CN | US | SS | VYVGART |
| 216146323 | 20230927 |  |  |  | Y | CN | US | SS | VYVGART |
| 217469057 | 20240321 |  |  |  | Y | CN | US | PS | VYVGART |
| 217469057 | 20240321 |  |  |  | Y | CN | US | SS | VYVGART |
| 219262594 | 20230617 | 81 | YR | M | Y | CN | US | PS | EFGARTIGIMOD ALFA-FCAB |
| 219262594 | 20230617 | 81 | YR | M | Y | CN | US | SS | EFGARTIGIMOD ALFA-FCAB |
| 219262594 | 20230617 | 81 | YR | M | Y | CN | US | SS | EFGARTIGIMOD ALFA-FCAB |
| 219346949 | 20231107 |  |  |  | Y | HP | US | PS | VYVGART |
| 219346949 | 20231107 |  |  |  | Y | HP | US | SS | VYVGART |
| 219346949 | 20231107 |  |  |  | Y | HP | US | SS | VYVGART |
| 219346949 | 20231107 |  |  |  | Y | HP | US | SS | VYVGART |
| 219346949 | 20231107 |  |  |  | Y | HP | US | SS | VYVGART |
| 219346949 | 20231107 |  |  |  | Y | HP | US | SS | VYVGART |
| 219346949 | 20231107 |  |  |  | Y | HP | US | SS | VYVGART |
| 219346949 | 20231107 |  |  |  | Y | HP | US | C | PREDNISONE |
| 219346949 | 20231107 |  |  |  | Y | HP | US | C | MESTINON |
| 219346949 | 20231107 |  |  |  | Y | HP | US | C | IVIGLOB EX |
| 219346949 | 20231107 |  |  |  | Y | HP | US | C | DIPHENHYDRAMINE HYDROCHLORIDE |
| 219346949 | 20231107 |  |  |  | Y | HP | US | C | ACETAMINOPHEN\HYDROCODONE |
| 219346949 | 20231107 |  |  |  | Y | HP | US | C | SODIUM CHLORIDE |
| 219346949 | 20231107 |  |  |  | Y | HP | US | C | EPINEPHRINE |
| 219346949 | 20231107 |  |  |  | Y | HP | US | C | ESTRACE |
| 219346949 | 20231107 |  |  |  | Y | HP | US | C | SODIUM CHLORIDE |
| 219346949 | 20231107 |  |  |  | Y | HP | US | C | NORTRIPTYLINE HYDROCHLORIDE |
| 219346949 | 20231107 |  |  |  | Y | HP | US | C | ALPRAZOLAM |
| 219346949 | 20231107 |  |  |  | Y | HP | US | C | HEPARIN |
| 219346949 | 20231107 |  |  |  | Y | HP | US | C | IMITREX |
| 219346949 | 20231107 |  |  |  | Y | HP | US | C | SERTRALINE HYDROCHLORIDE |
| 219346949 | 20231107 |  |  |  | Y | HP | US | C | OMEPRAZOLE |
| 219346949 | 20231107 |  |  |  | Y | HP | US | C | SOLIFENACIN SUCCINATE |
| 219346949 | 20231107 |  |  |  | Y | HP | US | C | METOPROLOL SUCCINATE |
| 219346949 | 20231107 |  |  |  | Y | HP | US | C | DIPHENHYDRAMINE |
| 219346949 | 20231107 |  |  |  | Y | HP | US | C | NORTRIPTYLINE |
| 219346949 | 20231107 |  |  |  | Y | HP | US | C | SERTRALINE |
| 219613758 | 20240314 |  |  |  | Y | CN | US | PS | VYVGART |
| 219613758 | 20240314 |  |  |  | Y | CN | US | SS | VYVGART |
| 219613758 | 20240314 |  |  |  | Y | CN | US | C | MESTINON |
| 219613758 | 20240314 |  |  |  | Y | CN | US | C | MESTINON |
| 219613758 | 20240314 |  |  |  | Y | CN | US | C | VITAMIN D |
| 219613758 | 20240314 |  |  |  | Y | CN | US | C | LIPITOR |
| 219613758 | 20240314 |  |  |  | Y | CN | US | C | OXYBUTYNIN |
| 219613758 | 20240314 |  |  |  | Y | CN | US | C | VITAMIN C |
| 219613758 | 20240314 |  |  |  | Y | CN | US | C | VITAMIN B12 |
| 219613758 | 20240314 |  |  |  | Y | CN | US | C | VITAMIN D3 |
| 219613758 | 20240314 |  |  |  | Y | CN | US | C | ASPIRIN |
| 219613758 | 20240314 |  |  |  | Y | CN | US | C | PRAVASTATIN |
| 219613758 | 20240314 |  |  |  | Y | CN | US | C | HYDROCHLOROTHIAZIDE |
| 219613758 | 20240314 |  |  |  | Y | CN | US | C | LOSARTAN |
| 219613758 | 20240314 |  |  |  | Y | CN | US | C | PREDNISONE |
| 221143421 | 20230320 |  |  |  | Y | CN | US | PS | VYVGART |
| 221951353 | 20230926 |  |  |  | Y | CN | US | PS | VYVGART |
| 221951353 | 20230926 |  |  |  | Y | CN | US | SS | VYVGART |
| 221951353 | 20230926 |  |  |  | Y | CN | US | C | ALPHA LIPOIC ACID |
| 221951353 | 20230926 |  |  |  | Y | CN | US | C | LEVOMEFOLIC ACID |
| 221951353 | 20230926 |  |  |  | Y | CN | US | C | PROBIOTICS NOS |
| 221951353 | 20230926 |  |  |  | Y | CN | US | C | DIETARY SUPPLEMENT\RESVERATROL |
| 221951353 | 20230926 |  |  |  | Y | CN | US | C | HERBALS\TURMERIC |
| 221951353 | 20230926 |  |  |  | Y | CN | US | C | ZINC |
| 221951353 | 20230926 |  |  |  | Y | CN | US | C | ZYRTEC |
| 221951353 | 20230926 |  |  |  | Y | CN | US | C | BIOTIN |
| 221951353 | 20230926 |  |  |  | Y | CN | US | C | Vi c |
| 221951353 | 20230926 |  |  |  | Y | CN | US | C | VALACYCLOVIR |
| 221951353 | 20230926 |  |  |  | Y | CN | US | C | LATANOPROST |
| 221951353 | 20230926 |  |  |  | Y | CN | US | C | Viactiv |
| 221951353 | 20230926 |  |  |  | Y | CN | US | C | DIETARY SUPPLEMENT |
| 221951353 | 20230926 |  |  |  | Y | CN | US | C | HYDROXYCHLOROQUINE |
| 221951353 | 20230926 |  |  |  | Y | CN | US | C | SOLIRIS |
| 221951353 | 20230926 |  |  |  | Y | CN | US | C | AZATHIOPRINE |
| 221951353 | 20230926 |  |  |  | Y | CN | US | C | PREDNISONE |
| 221951353 | 20230926 |  |  |  | Y | CN | US | C | ONDANSETRON HYDROCHLORIDE |
| 221951353 | 20230926 |  |  |  | Y | CN | US | C | MELOXICAM |
| 221951353 | 20230926 |  |  |  | Y | CN | US | C | OMEPRAZOLE |
| 222410453 | 20230707 |  |  |  | Y | CN | US | PS | VYVGART |
| 222564495 | 20240626 |  |  |  | Y | CN | US | PS | EFGARTIGIMOD ALFA-FCAB |
| 222564495 | 20240626 |  |  |  | Y | CN | US | SS | EFGARTIGIMOD ALFA-FCAB |
| 222564495 | 20240626 |  |  |  | Y | CN | US | SS | EFGARTIGIMOD ALFA-FCAB |
| 222564495 | 20240626 |  |  |  | Y | CN | US | SS | EFGARTIGIMOD ALFA-FCAB |
| 222692562 | 20230511 |  |  |  | Y | CN | US | PS | VYVGART |
| 222821303 | 20230804 |  |  |  | Y | CN | US | PS | EFGARTIGIMOD ALFA-FCAB |
| 222821303 | 20230804 |  |  |  | Y | CN | US | SS | EFGARTIGIMOD ALFA-FCAB |
| 224003571 | 20230602 |  |  |  | Y | HP | US | PS | VYVGART |
| 225395375 | 20240412 |  |  |  | Y | CN | US | PS | VYVGART |
| 225395375 | 20240412 |  |  |  | Y | CN | US | SS | VYVGART |
| 226548624 | 20231123 |  |  |  | Y | CN | US | PS | VYVGART |
| 226548624 | 20231123 |  |  |  | Y | CN | US | SS | VYVGART |
| 226548624 | 20231123 |  |  |  | Y | CN | US | C | IRON |
| 226548624 | 20231123 |  |  |  | Y | CN | US | C | B12 1000 SR |
| 227430486 | 20240307 |  |  |  | Y | CN | US | PS | VYVGART |
| 228376694 | 20240314 |  |  |  | Y | CN | US | PS | EFGARTIGIMOD ALFA-FCAB |
| 228382453 | 20240423 |  |  |  | Y | CN | US | PS | VYVGART |
| 228511429 | 20240314 |  |  |  | Y | CN | US | PS | VYVGART |
| 228511429 | 20240314 |  |  |  | Y | CN | US | SS | VYVGART |
| 228511429 | 20240314 |  |  |  | Y | CN | US | SS | VYVGART |
| 228511429 | 20240314 |  |  |  | Y | CN | US | SS | VYVGART |
| 228511429 | 20240314 |  |  |  | Y | CN | US | SS | VYVGART |
| 228511429 | 20240314 |  |  |  | Y | CN | US | SS | VYVGART |
| 228511429 | 20240314 |  |  |  | Y | CN | US | C | DIPHENHYDRAMINE HYDROCHLORIDE |
| 228511429 | 20240314 |  |  |  | Y | CN | US | C | EPINEPHRINE |
| 228511429 | 20240314 |  |  |  | Y | CN | US | C | SODIUM CHLORIDE |
| 228511429 | 20240314 |  |  |  | Y | CN | US | C | PREDNISONE |
| 228511429 | 20240314 |  |  |  | Y | CN | US | C | FAMOTIDINE |
| 228511429 | 20240314 |  |  |  | Y | CN | US | C | LAMOTRIGINE |
| 228511429 | 20240314 |  |  |  | Y | CN | US | C | SODIUM CHLORIDE |
| 228511429 | 20240314 |  |  |  | Y | CN | US | C | ONDANSETRON |
| 228511429 | 20240314 |  |  |  | Y | CN | US | C | VITAMINS |
| 229116051 | 20230906 |  |  |  | Y | CN | US | PS | VYVGART |
| 229796232 | 20231025 |  |  |  | Y | CN | US | PS | VYVGART |
| 229796232 | 20231025 |  |  |  | Y | CN | US | C | LORAZEPAM |
| 229796232 | 20231025 |  |  |  | Y | CN | US | C | MORPHINE |
| 230339832 | 20231121 |  |  |  | Y | CN | US | PS | VYVGART |
| 230339832 | 20231121 |  |  |  | Y | CN | US | SS | VYVGART |
| 230339832 | 20231121 |  |  |  | Y | CN | US | SS | VYVGART |
| 230339832 | 20231121 |  |  |  | Y | CN | US | C | LEVOTHYROXINE |
| 230339832 | 20231121 |  |  |  | Y | CN | US | C | PREDNISONE |
| 230339832 | 20231121 |  |  |  | Y | CN | US | C | MYCOPHENOLATE MOFETIL |
| 230339832 | 20231121 |  |  |  | Y | CN | US | C | DULOXETINE HYDROCHLORIDE |
| 230339832 | 20231121 |  |  |  | Y | CN | US | C | SERTRALINE HYDROCHLORIDE |
| 230339832 | 20231121 |  |  |  | Y | CN | US | C | PYRIDOSTIGMINE BROMIDE |
| 230339832 | 20231121 |  |  |  | Y | CN | US | C | LOPERAMIDE |
| 230339832 | 20231121 |  |  |  | Y | CN | US | C | ALENDRONATE SODIUM |
| 231250016 | 20240503 |  |  |  | Y | CN | US | PS | VYVGART |
| 231250016 | 20240503 |  |  |  | Y | CN | US | SS | VYVGART |
| 231250016 | 20240503 |  |  |  | Y | CN | US | SS | VYVGART |
| 231250016 | 20240503 |  |  |  | Y | CN | US | SS | VYVGART |
| 231250016 | 20240503 |  |  |  | Y | CN | US | SS | VYVGART |
| 231250016 | 20240503 |  |  |  | Y | CN | US | SS | VYVGART |
| 231250016 | 20240503 |  |  |  | Y | CN | US | C | SODIUM CHLORIDE |
| 231250016 | 20240503 |  |  |  | Y | CN | US | C | SODIUM CHLORIDE |
| 231250016 | 20240503 |  |  |  | Y | CN | US | C | EPINEPHRINE |
| 231250016 | 20240503 |  |  |  | Y | CN | US | C | HEPARIN |
| 232840632 | 20240311 |  |  |  | Y | CN | US | PS | VYVGART |
| 232923573 | 20240328 |  |  |  | Y | CN | US | PS | VYVGART |
| 232923573 | 20240328 |  |  |  | Y | CN | US | SS | VYVGART |
| 232923573 | 20240328 |  |  |  | Y | CN | US | SS | VYVGART |
| 232923573 | 20240328 |  |  |  | Y | CN | US | SS | PREDNISONE |
| 232923573 | 20240328 |  |  |  | Y | CN | US | SS | CELLCEPT |
| 233421751 | 20231227 | 76 | YR | M | Y | CN | US | PS | VYVGART |
| 233508561 | 20231229 |  |  | F | Y | CN | US | PS | VYVGART |
| 233752992 | 20240118 |  |  | F | Y | CN | US | PS | VYVGART |
| 233753011 | 20240106 |  |  | M | Y | CN | US | PS | VYVGART |
| 234247641 | 20240120 |  |  | F | Y | CN | US | PS | VYVGART |
| 234468051 | 20240126 |  |  | M | Y | CN | US | PS | VYVGART |
| 234468191 | 20240126 |  |  | F | Y | CN | US | PS | VYVGART |
| 234793021 | 20240205 |  |  |  | Y |  | US | PS | VYVGART |
| 234946351 | 20240207 |  |  | F | Y | CN | US | PS | VYVGART |
| 235291345 | 20240509 |  |  |  | Y | CN | US | PS | VYVGART |
| 235300542 | 20240416 |  |  |  | Y | CN | US | PS | VYVGART |
| 235300542 | 20240416 |  |  |  | Y | CN | US | C | HUMAN IMMUNOGLOBULIN G |
| 235301231 | 20240216 |  |  |  | Y | CN | US | PS | VYVGART |
| 235419562 | 20240607 |  |  |  | Y | CN | US | PS | VYVGART |
| 235486407 | 20240513 |  |  |  | Y | CN | US | PS | VYVGART |
| 235486407 | 20240513 |  |  |  | Y | CN | US | SS | VYVGART |
| 235786791 | 20240229 |  |  |  | Y | CN | US | PS | VYVGART |
| 235786791 | 20240229 |  |  |  | Y | CN | US | SS | VYVGART |
| 235790681 | 20240229 |  |  |  | Y | CN | US | PS | VYVGART |
| 236327762 | 20240502 |  |  |  | Y | CN | US | PS | VYVGART HYTRULO |
| 236327762 | 20240502 |  |  |  | Y | CN | US | SS | VYVGART |
| 236328061 | 20240314 |  |  |  | Y | CN | US | PS | VYVGART |
| 236328212 | 20240422 |  |  |  | Y | CN | US | PS | VYVGART |
| 236328293 | 20240530 |  |  |  | Y | CN | US | PS | VYVGART |
| 236328293 | 20240530 |  |  |  | Y | CN | US | SS | VYVGART |
| 236328293 | 20240530 |  |  |  | Y | CN | US | C | EPINEPHRINE |
| 236328293 | 20240530 |  |  |  | Y | CN | US | C | SODIUM CHLORIDE |
| 236328293 | 20240530 |  |  |  | Y | CN | US | C | OMEPRAZOLE |
| 236328293 | 20240530 |  |  |  | Y | CN | US | C | PAROXETINE HYDROCHLORIDE |
| 236328293 | 20240530 |  |  |  | Y | CN | US | C | TAMSULOSIN HYDROCHLORIDE |
| 236328293 | 20240530 |  |  |  | Y | CN | US | C | SODIUM CHLORIDE |
| 236328293 | 20240530 |  |  |  | Y | CN | US | C | LASIX |
| 236328293 | 20240530 |  |  |  | Y | CN | US | C | PFIZER-BIONTECH COVID-19 VACCINE |
| 236328293 | 20240530 |  |  |  | Y | CN | US | C | COZAAR |
| 236328293 | 20240530 |  |  |  | Y | CN | US | C | GABAPENTIN |
| 236328293 | 20240530 |  |  |  | Y | CN | US | C | ROSUVASTATIN |
| 236328293 | 20240530 |  |  |  | Y | CN | US | C | CALCIUM |
| 236328293 | 20240530 |  |  |  | Y | CN | US | C | BUPROPION |
| 236328293 | 20240530 |  |  |  | Y | CN | US | C | SYNTHROID |
| 236328293 | 20240530 |  |  |  | Y | CN | US | C | HEPARIN |
| 236328293 | 20240530 |  |  |  | Y | CN | US | C | MESTINON |
| 236328293 | 20240530 |  |  |  | Y | CN | US | C | METOPROLOL SUCCINATE |
| 236328293 | 20240530 |  |  |  | Y | CN | US | C | PROAIR HFA |
| 236328293 | 20240530 |  |  |  | Y | CN | US | C | WEGOVY |
| 236328293 | 20240530 |  |  |  | Y | CN | US | C | MONTELUKAST SODIUM |
| 236328293 | 20240530 |  |  |  | Y | CN | US | C | PREDNISONE |
| 236328293 | 20240530 |  |  |  | Y | CN | US | C | ASPIRIN |
| 236328293 | 20240530 |  |  |  | Y | CN | US | C | FOLIC ACID\IRON |
| 236328293 | 20240530 |  |  |  | Y | CN | US | C | DIPHENHYDRAMINE HYDROCHLORIDE |
| 236328293 | 20240530 |  |  |  | Y | CN | US | C | ACETAMINOPHEN |
| 236328293 | 20240530 |  |  |  | Y | CN | US | C | SHINGRIX |
| 236328293 | 20240530 |  |  |  | Y | CN | US | C | PNEUMOVAX 23 |
| 236328293 | 20240530 |  |  |  | Y | CN | US | C | VITAMIN D3 |
| 236328293 | 20240530 |  |  |  | Y | CN | US | C | SYMBICORT |
| 236466041 | 20240319 |  |  |  | Y | CN | US | PS | VYVGART |
| 237618281 | 20240419 |  |  |  | Y | CN | US | PS | VYVGART |
| 237935963 | 20240529 |  |  |  | Y | PH | US | PS | VYVGART |
| 237935963 | 20240529 |  |  |  | Y | PH | US | C | MESTINON |
| 238388032 | 20240610 |  |  |  | Y | CN | US | PS | VYVGART HYTRULO |
| 238495692 | 20240521 |  |  |  | Y | CN | US | PS | VYVGART |
| 238639411 | 20240516 |  |  |  | Y | CN | US | PS | VYVGART |
| 238825251 | 20240522 |  |  |  | Y | CN | US | PS | VYVGART |
| 238825251 | 20240522 |  |  |  | Y | CN | US | SS | VYVGART HYTRULO |
| 239046741 | 20240527 |  |  |  | Y | CN | US | PS | VYVGART |
| 239046741 | 20240527 |  |  |  | Y | CN | US | SS | POTASSIUM |
| 239046741 | 20240527 |  |  |  | Y | CN | US | C | PRAVASTATIN SODIUM |
| 239046741 | 20240527 |  |  |  | Y | CN | US | C | GLIPIZIDE |
| 239046741 | 20240527 |  |  |  | Y | CN | US | C | ROSUVASTATIN CALCIUM |
| 239046741 | 20240527 |  |  |  | Y | CN | US | C | SPIRONOLACTONE |
| 239046741 | 20240527 |  |  |  | Y | CN | US | C | PENICILLIN V POTASSIUM |
| 239046741 | 20240527 |  |  |  | Y | CN | US | C | ATORVASTATIN CALCIUM |
| 239046741 | 20240527 |  |  |  | Y | CN | US | C | AMITRIPTYLINE HYDROCHLORIDE |
| 239046741 | 20240527 |  |  |  | Y | CN | US | C | EZETIMIBE |
| 239049731 | 20240527 |  |  |  | Y | CN | US | PS | VYVGART |
| 239049731 | 20240527 |  |  |  | Y | CN | US | C | BACTRIM |
| 239147251 | 20240529 |  |  |  | Y | CN | US | PS | VYVGART HYTRULO |
| 239193452 | 20240606 |  |  |  | Y | CN | US | PS | VYVGART |
| 239193452 | 20240606 |  |  |  | Y | CN | US | C | PREDNISONE |
| 239193452 | 20240606 |  |  |  | Y | CN | US | C | MESTINON |
| 239483281 | 20240607 |  |  |  | Y | CN | US | PS | VYVGART |
| 239483281 | 20240607 |  |  |  | Y | CN | US | C | PREDNISONE |
